# Plant-based vaccines against viruses

**DOI:** 10.1186/s12985-014-0205-0

**Published:** 2014-12-03

**Authors:** Edward P Rybicki

**Affiliations:** Biopharming Research Unit, Department of Molecular & Cell Biology and Institute of Infectious Disease and Molecular Medicine, University of Cape Town, Private Bag X3, Rondebosch, 7701 Cape Town, South Africa

**Keywords:** Virus, Vaccine, Biofarming, Plant-made antigen, Monoclonal antibody, HIV, HBV, HCV, HPV, Influenza, Bluetongue, Rabies, Ebola, ZMapp

## Abstract

Plant-made or “biofarmed” viral vaccines are some of the earliest products of the technology of plant molecular farming, and remain some of the brightest prospects for the success of this field. Proofs of principle and of efficacy exist for many candidate viral veterinary vaccines; the use of plant-made viral antigens and of monoclonal antibodies for therapy of animal and even human viral disease is also well established. This review explores some of the more prominent recent advances in the biofarming of viral vaccines and therapies, including the recent use of ZMapp for Ebolavirus infection, and explores some possible future applications of the technology.

## Introduction

The science of “molecular farming”, or the use of plants and plant cell cultures to produce high-value recombinant proteins, started with the production via transgenic tobacco and sunflower of chimaeric human growth hormone in 1986 [1], then of monoclonal antibodies in transgenic tobacco in 1989 [2], and human serum albumin in transgenic tobacco and cell cultures [3]. This was followed from 1992 onwards with the expression of the first candidate virus vaccine antigens: these were Hepatitis B virus (HBV) surface antigen (HBsAg) in 1992 [4], and a VP1 epitope of foot-and-mouth disease virus (FMDV) expressed on the surface of particles of recombinant Cowpea mosaic virus (CPMV) in 1993 ([5] see Table 1).

**Table 1:** **Highlights of historical and recent activity in virus vaccine biofarming**

**Virus and vaccine candidate**	**Reference**
**Hepatitis B virus**	
First expression of HBV surface antigen in plants	[4]
Human clinical trial of plant-produced HBsAg	[16]
First production of HBsAg in transgenic banana	[17]
Use of rTMV-mediated transient expression to produce ~300 mg/kg HBsAg	[20]
Use of rBeYDV-mediated transient expression to produce 800 mg/kg HBc Ag	[24]
Tabletised lyophilised transgenic lettuce containing HBsAg VLPs is orally immunogenic in mice	[19]
**Hepatitis C virus**	
Use of rTMV to express R9 epitope of E2 protein as CTB fusion	[30]
Expression of chimaeric plant virus coat protein molecules containing R9	[31-33]
R9-CMV CP VLPs orally immunogenic via feeding of lettuce leaves in rabbits	[34]
Mixed Th1/Th2 response in mice to rodlike PMV-E2 epitope chimaeric VLPs	[36]
Co-expression of whole E2 and calnexin and calreticulin increases E2 accumulation in plants	[37]
**Influenza viruses**	
Evidence of high-yield expression of H5 haemagglutinin-derived VLPs via transient expression	[44]
10 million vaccine dose “rapid fire” milestone by Medicago Inc. in DARPA Blue Angel programme	[50]
Human clinical trial of plant-made H5N1 vaccine candidate	[47]
HA-only VLPs produced for H7N9 outbreak virus	[54]
Phase 1 trials of H1N1pdm and HPAI H5N1 HA-derived plant-made products	[48]
Testing of plant-made engineered soluble trimeric HA (H1N1pdm) in mice	[58]
Adjuvanting of monomeric H1N1pdm HA with SiO_2_ and bis-(3′,5′)-cyclic dimeric guanosine monophosphate (c-di-GMP)	[59]
Emergency response influenza vaccine candidates made in South Africa	[43]
Conjugation of plant-made HA to TMV particles and successful testing in mice	[60]
Elicitation of neutralising Ab with elastin-like polypeptide fused with stabilised soluble trimer-forming H5N1 HA	[61]
Presentation of M2e epitope on surface of rTMV virions elicits protective immunity to homologous and heterologous challenge in mice	[64]
**Papillomaviruses**	
Proof of efficacy of a plant-made Cottontail rabbit and Rabbit oral papillomavirus vaccines	[74,75]
High yields of HPV-16 L1 and VLPs via agroinfiltration-mediated transient expression or via transplastomic expression	[76,77]
Transplastomic expression of capsomere-forming HPV-16 L1 fused with *Escherichia coli* LTB as a built-in adjuvant	[78]
Successful expression of HPV-8 and Bovine papillomavirus L1 VLPs in plants	[79,80]
Co-expression of HPV-16 L1 with *E coli* LTB and oral immunisation elicits increased intestinal mucosal IgA responses to L1	[90]
High-yield plant transient expression of chimaeric L1::L2 VLPs and proof of increased breadth of immune response	[91]
rPVX CP fusion with L2_108–120_ epitope yields well and elicits high-titre anti-L2 protein sera in mice	[97]
Plant production, scale-up and protective efficacy in mouse model of therapeutic E7GGG-LicKM fusion protein vaccine	[85-87]
Plant expressed HPV-16 L1 with C-terminal string of E6 and E7 T-cell epitopes is viable prophylactic/therapeutic vaccine candidate	[98]
Production and proof of efficacy in mice of soluble E7GGG therapeutic vaccine in transplastomic *Chlamydomonas reinhardtii*	[100]
Proof of yield increase and efficacy in a mouse tumour model of shuffled E7 protein fused to Zera® peptide	[102]
**Human immunodeficiency virus**	
HIV-1 p24 capsid protein expressed successfully in transgenic tobacco	[107]
Transgenic maize as production platform for oral vaccine delivery tested using SIV major surface glycoprotein gp130	[108]
Transiently-expressed gp41-derived molecule fused to CTB elicits anti-membrane proximal region (MPR) antibodies in mice	[109,110]
Gag-derived antigens expressed transiently and transgenically as CTL-inducing booster immunogens	[113]
High-yield transplastomic expression of Gag VLPs	[117]
Phase I clinical trial of anti-HIV MAbs produced in transgenic tobacco	[116]
rTMV-based expression of Gag VLP-based deconstructed multiantigens incorporating gp 41 MPER region	[124]
First successful published expression of HIV Env in plants: 89.6.P gp140ΔCFI envelope protein expressed at high yield	[125]
Transient expression of HIV Env-H5 HA fusion molecule results in VLP formation	[126]
**Veterinary and “One Health” vaccines**	
Production in plants and proof of efficacy in sheep of intact VLPs of Bluetongue virus	[128]
Rabies virus vaccines produced in plants and shown to be protective including after oral administration	[138-141]
Crimean-Congo haemorrhagic fever virus Gc and Gn glycoproteins immunogenic in mice via oral or parenteral administration	[144]
Plant-produced Rift Valley fever virus Gn and N proteins immunogenic after feeding mice transgenic *Arabidopsis*	[145]
Expression via rBeYDV and successful immunogenicity trial in mice of Ebola GP1/anti-GP1 MAb HC	[158]
**Anti-viral therapeutic antibodies**	
Production and successful efficacy testing of anti-Rabies virus MAbs made in transgenic tobacco	[147]
High-yield production via rBeYDV of anti-Ebolavirus Zaire and West Nile virus MAbs	[149,150]
Transient expression via rTMV, and successful pre- and post-exposure efficacy trial in macaques, of anti-Ebola MAb cocktail	[151,152]
Successful efficacy trial in macaques of the ZMApp therapeutic MAb cocktail	[153]

Legend: rTMV = recombinant Tobacco mosaic virus, including ICON vectors. rBeYDV = recombinant Bean yellow dwarf mastrevirus.

Initially, the prevailing idea for the use of plant-produced vaccines was that these should be delivered in plant material, as edible vaccines – something that was already being questioned as early as 1996 [6]. However, this concept has now largely fallen out of favour, with the realisation that administration of vaccines to humans requires standardisation of dose and some measure of quality control, and the necessity for purification and formulation has largely been accepted [7]. Over the years since 1989, then, many proteins have been expressed as candidate vaccines or therapeutics, and many different plant-based expression systems have been tried, with a growing trend towards transient expression systems based on infiltration of whole plants with recombinant *Agrobacterium tumefaciens* (agroinfiltration) and the use of “deconstructed” plant viral vectors (reviewed in [8]).

Virus vaccines have been a large and exciting part of this field almost from its beginning, for disease agents ranging from Hepatitis B to C to Foot and mouth disease viruses, from Human papillomavirus and Human rotavirus to ovine Bluetongue and Rabbit haemorrhagic disease viruses, to mention just a few. Aspects of this history have been covered recently, and in particular for virus-like particle based vaccines including rotaviruses and Norwalk virus [9], Human papillomaviruses [10] and Hepatitis B virus [11], and so these will not be discussed in detail here except where there is new material to be covered.

This review will cover the relevant recent history of virus-specific candidate vaccines and virus-specific therapeutic antibodies made in plants, with a view to providing object examples of successful approaches and especially of dual human/animal use or “One Health” examples (http://www.onehealthinitiative.com/), in order to help inform future work.

## Recent candidate viral vaccines produced in plants

### Hepatitis B virus vaccines

Hepatitis B virus (HBV) vaccines are one of the blockbuster vaccine success stories of modern times: since identification of the virus in the 1960s, it took less than 20 years for a subunit vaccine to get to market. However, this was in the form of 22 nm subviral particles purified from the serum of human carriers of HBV, and although highly effective, was expensive to produce and of limited supply – to say nothing of the ever-present risk associated with a blood product isolated from HBV carriers who may carry any number of other, as yet undetected viruses. It was a triumph of modern molecular biology, therefore, when a very similar virus-like particle (VLP) vaccine derived from expression of the HBV small surface antigen (S-HBsAg) was developed in 1984 [12]. While this was initially still expensive – US$40/dose, with three intramuscular doses being necessary – prices have come down very significantly, to the point that more than 110 countries now routinely immunise infants as part of the Extended Programme of Immunisation (EPI) [13]. The recombinant vaccines are highly effective and safe, and have helped set the standard for later introductions, such as of the recombinant VLP-based Human papillomavirus (HPV) vaccines.

However, there is still space for improvements in HBV vaccines, both in terms of cost of goods, and in specific antigen content. An increasing desire worldwide for “needle-free” vaccine delivery, for example, would require cheaper production of larger amounts of antigen for oral delivery, which current production modalities would not be able to meet. Problems with non-response of certain groups of people to the current vaccines have also necessitated the development of third generation products, containing the middle (M-HBsAg) and/or large (L-HBsAg) surface antigens, which contain the strongly immunogenic preS1 and/or preS2 domains. However, these vaccines are more expensive and less readily available [11]. Accordingly, plant production of HBsAg-based HBV vaccines has gone on for over 20 years, with a variety of products being made; pre-clinical testing of oral delivery of transgenic potato-delivered products for almost as long [14], with a preclinical trial of an orally-delivered product in 2001 [15], and human clinical trial in 2005 [16].

Oral delivery of HBsAg in transgenic plant material has not proved to be particularly effective, however, with immunogenicity generally being low [7]. This has effectively led to the general curtailment of the transgenic-plant-as vaccine efforts [9]. It is interesting in this regard that despite the concept of “vaccination via banana” having been hyped in the popular press since the 1990s (eg: http://www.theguardian.com/science/2000/sep/08/gm.infectiousdiseases), it was not until 2005 that HBsAg was first expressed in transgenic banana fruit in India, albeit at relatively low yield [17]. A recent review has proposed a combination of approaches, with parenteral vaccination with purified plant-produced HBsAg followed by oral boosting with less well purified antigen as tablets or capsules [18]: preliminary studies in mice using lyophilised HBsAg VLP-producing transgenic lettuce converted into tablets appear to add weight to the proposal [19].

The highest plant yield of conventional (=S, or small) HBsAg was achieved via use of deconstructed Tobacco mosaic virus-based cDNA MagnICON vectors (Icon Genetics, Halle, Germany): this was around 300 mg/kg wet weight in *Nicotiana benthamiana*, and the recombinant protein was full-length, formed disulphide-linked dimers, displayed the conformationally-determined ‘a’ antigenic determinant, and assembled correctly into VLPs. Interestingly, vacuum-mediated agroinfiltration of whole plants led to a better product, as determined by presence of the ‘a’ determinant [20].

Plant expression of HBV antigens other than the standard S HBsAg has been attempted in recent years by a number of groups. The Arizona Biodesign Institute group, who pioneered much of the biofarming of HBV vaccines, showed in 2004 that the S form of HBsAg could be used via transient agroinfiltration-mediated expression as a useful fusion partner, with an N-terminal fusions of up to 239 amino acids being tolerated without alteration of its particle-forming ability or major antigenic properties [21]. They followed this with the demonstration that the HBsAg middle protein (M protein) - with the highly immunogenic 55 amino acid pre-S2 region fused at N-terminus of the S protein – could be successfully produced in plants, and elicited stronger humoral immune responses than the S protein when injected into mice [22]. The same group used MagnICON vectors to express over 2 g/kg wet weight of plants of VLP-forming and highly immunogenic HBcAg, the “core” antigen [23]: HBc has considerable potential both as a therapeutic vaccine and as a display vehicle for other peptides, including the HBV preS region [11]. A subsequent paper from the Biodesign Institute group detailed the use of a ssDNA Bean yellow dwarf mastrevirus-based vector system (reviewed here [24]) to produce 800 mg/kg of HBcAg in *N benthamiana* [25].

It is safe to say that the potential for production of HBV vaccines in plants has been well realised: while wholly edible/orally-administered vaccines may remain a pipedream, levels of antigen production for both HBsAg and HBcAg have been achieved that are some of the highest for any plant-produced molecules, and the products have been shown to be antigenically appropriate and highly immunogenic.

### Hepatitis C vaccines

Between 130 and 150 million people worldwide are chronically infected with Hepatitis C virus (HCV), and around 350 000 people die annually from HCV-related liver disease [26]. While a significant proportion of those infected will clear their infections naturally, the majority will go on to develop chronic infections – with a 15-30% risk of cirrhosis of the liver within 20 years. The only treatment presently available is antivirals, with the most common regime being combination antiviral therapy with interferon and ribavirin, which are effective against all viral genotypes. Newer drugs are becoming available, such as the very promising sofosbuvir [27]; however, vaccines will be the only effective means of preventing infection from occurring.

Plant production of candidate HCV vaccines has a reasonably long history, given that the virus was only described in 1989 [28,29]. It is interesting that the first products have all been intended as therapeutic vaccines: in 2000, Nemchinov and colleagues described the use of a Tobacco mosaic virus (TMV)-derived vector in *N benthamiana* to express a synthetic hypervariable region 1 (HVR1)-derived peptide called R9, a potential neutralising epitope of HCV derived from the envelope protein E2, fused to the C-terminal of the B subunit of cholera toxin (CTB). The plant-derived HVR1/CTB reacted with immune sera from individuals infected with four of the major genotypes of HCV, and mice immunised intranasally with crude plant extract produced anti-HVR1 antibodies which specifically bound HCV VLPs [30]. This was followed by a succession of reports on plant expression of chimaeric plant virus coat protein molecules containing the same (R9) epitope: these included the use of Cucumber mosaic virus (CMV) CP, with immunoreactivity of sera from chronically infected patients with the recombinant CP [31] leading to use of purified R9-CMV to elicit in vivo responses in rabbits, and in vitro cellular responses as measured by interferon gamma production for lymphocytes from human patients [32,33]. Subsequently, the same group showed that purified R9-CMV particles were stable under simulated gastric and intestinal conditions, and could elicit a humoral immune response in rabbits fed with R9-CMV infected lettuce plants [34].

The R9 epitope has also been expressed as a fusion product with the Alfalfa mosaic virus (AMV) CP, via transfecting plants transgenic for AMV RNAs 1 and 2 with recombinant CP-encoding RNA3: the R9/ALMV-CP reacted with HVR1-specific monoclonal antibodies and immune sera from individuals infected with HCV [35].

In another approach, use of an E2-derived epitope as a plant-produced C-terminal fusion to Papaya mosaic virus (PMV) CP led to formation of flexible rodlike VLPs which were actively internalised in bone-marrow-derived antigen presenting cells (APCs). C3H/HeJ mice injected twice with the VLPs exhibited a humoral response lasting more than 120 days against both the CP and the E2 epitope, with the production of IgG1, IgG2a, IgG2b and IgG3 suggesting a Th1/Th2 mixed response [36].

Another interesting recent development was the filing of a USPTO patent application on plant production of the whole E2 protein, as apparently this has not been particularly successful because of misfolding. The patent covers agroinfecting a *N. benthamiana* cell expressing the recombinant E2 protein with one or both of the molecular chaperones calnexin and calreticulin, as this leads to an improvement in the yield of recombinant E2 protein [37]. This adds to what appears to be a successful expression, albeit at low level, of HCV E1 protein in plants [38].

It appears as though plant production of candidate therapeutic vaccines for HCV is quite well covered; however, the prophylactic vaccine space is far less well investigated: hopefully, this will change with optimisation of plant production of full-length E2 protein as detailed above.

### Influenza virus vaccines

Human seasonal influenza is currently mainly caused by viruses from two distinct genera of *Influenzavirus*: these are three Influenza A viruses (H1N1, H1N1pdm09, and H3N2), and two lineages of Influenza B virus (Yamagata and Victoria). Annual attack rates globally are estimated at between 5–10% in adults and 20–30% in children, with about 3–5 million cases of severe illness, and about 250 000–500 000 deaths [39,40]. The conventional chicken egg-based inactivated whole-virus split vaccine technology has a production capacity as of 2011 of 1.42 billion doses of trivalent vaccine, and a production level of 620 million doses - albeit with a six-month lead-in period every year [41]. This is manifestly obviously not capable of dealing with pandemics, when

“…the potential vaccine supply would fall several billion doses short of the amount needed to provide protection to the global population” [42].

It is worth noting that this report was issued just three years before the 2009 pandemic – and was prophetic in its predictions of vaccine shortages, and of the overly long interval between identification of the pandemic agent, and availability of vaccines. The report went on to say:

1. A vaccine cannot be developed with certainty until after the pandemic virus emerges.
2. Current global capacity to manufacture influenza vaccines is limited.
3. Two doses may be needed for a pandemic vaccine because of the absence of pre-existing immunity. This could further delay time to achieve protection and add operational challenges to delivery.
4. **High antigen content may be required; this would limit the total number of doses that can be made available with the current egg-based technology for inactivated vaccines**. [my emphasis]
5. The target population for vaccination could potentially be the entire global population of over six billion; this would require comprehensive resources to support operational and logistic demands in many countries.

In respect of the last point, it is worth noting that in 2001, while northern hemisphere influenza vaccine production capacity was rated at 1,069 million doses, with actual production of 534 million doses, southern hemisphere capacity was 352 million doses, with a production of only 86 million [41]. Thus, provision of pandemic vaccines for the “Global South” would be - and was in fact, in 2009 [43] - gravely lacking with current technology. I am indebted to one of the referees of this review for the observation that “It is important that all the eggs are literally not placed in one basket – or all of the vaccines put in eggs”.

Perhaps fortunately, then, influenza vaccines have been the major success story for plant-expressed antigens, largely due to two major factors: first, the haemagglutinin (HA) protein is the main determinant of neutralisation, and the only essential component of a vaccine; second, it appears to be well expressed in plants, and to fold properly. Perhaps the most significant factor of plant expression of HA, however, is the fact that expression of this alone is sufficient for the efficient formation at high yield of highly immunogenic VLPs, which bud from the plant cell outer membrane [44]. This is especially relevant to influenza vaccine development in light of the fact that HA-containing VLPs produced in insect cells elicit broader cross-neutralising immune responses than whole virion inactivated influenza virus or recombinant HA [45]. These properties have been exploited by a number of groups in work reported on in detail elsewhere [46-48]; accordingly, this review will be limited to recent developments of specific interest to pandemic and seasonal human vaccine development.

Plant-based technology lends itself very well to “orphan” or “niche” vaccines, because of what is in effect infinite scalability of production. It is also very well suited for manufacture of “rapid response” vaccines, such as those directed against pandemic influenza and bioterror agents: for this reason the US Defense Advanced Research Projects Agency (DARPA) in 2005 launched an Accelerated Manufacturing of Pharmaceuticals (AMP) programme that included an investigation of the suitability of plants as a manufacturing platform for the purpose. In 2009, as a response to the H1N1 “swine flu” virus pandemic, they initiated the “Blue Angel” effort, which was:

**“…an accelerated and integrated effort to deliver effective interventions for pandemic influenza”**

Part of this involved using US$100 million to fund a challenge for four companies to demonstrate a capability to use plants to produce 100 million doses of influenza vaccine a month. These were the Fraunhofer USA Center for Molecular Biotechnology in Delaware, Kentucky Bioprocessing in Owensboro, the Project GreenVax consortium with partners from Texas A&M University system and G-Con from Texas, and Medicago USA in North Carolina [49]. As of July 2012, Medicago Inc. had produced, as part of a “rapid fire” milestone, more than 10 million doses of an H1N1 VLP-based influenza vaccine candidate in one month, by Phase 1-appropriate current good manufacturing practices (cGMP) [50].

Latest developments from contenders in this challenge include preclinical and human clinical trials of a number of HA-based products. The first was of Medicago’s H5N1 A/Indonesia/5/05 HA VLP vaccine candidate, which constituted “…the first ever report of administration of a plant-made VLP vaccine to humans” [47]. In preclinical work reported in this paper, two low doses of Alhydrogel® alum-adjuvanted plant-made VLPs (1.8 μg) in ferrets prevented pathology and reduced viral loads following heterotypic (A/Vietnam/1203/04 H5N1 clade 1 virus) lethal challenge. Interestingly, this protection occurred despite the fact that HAI titres to the A/Vietnam/1203/04 challenge virus were detectable in only 75–87.5% of the challenged ferrets: this prompted the comment that the correlates of protection for influenza virus infection are not fully understood, and that the VLPs are probably stimulating innate immune responses not seen for conventional vaccines.

The human trial of the H5 HA VLPs was performed in healthy adults 18–60 years of age who received 2 doses 21 days apart of 5, 10 or 20 mg of alum-adjuvanted H5 VLP vaccine or placebo (alum). Immunogenicity was evaluated using Haemagglutination-Inhibition (HAI), Single Radial Hemolysis (SRH) and MicroNeutralisation (MN) assays: results from all three assays were highly correlated, with clear dose-responses with all measures of immunogenicity. Almost 96% of those in the 2×10 or 2×20 μg dose groups mounted detectable MN responses, indicating promising immunogenicity. In their words, “These data are particularly encouraging in light of the fact that traditional, egg-based split H5N1 vaccines showed only modest immunogenicity in humans at doses as high as 45 mg with or without alum as an adjuvant”.

One particularly encouraging aspect of this study was their testing of plant-specific glycan responses in the study group. One of the purported potential hazards of plant-made vaccines is the possibility that they might exacerbate pre-existing allergies to plant N-glycans, or even elicit such responses de novo [51]. In a clear negation of this potential for this vaccine, this study found no IgEs to plant-specific glycans in any of the 48 subjects, and no significant statistical difference in increase of IgGs to plant glycans between placebo and vaccine groups. That this may be important in influenza vaccines made in different expression systems is shown by evidence that glycosylation of recombinant A/California/04/09 HA made in insect cells and plants is different, with high mannose type glycans in plant-expressed HAs, and complex type glycoforms for the insect-expressed HA [52]. Further evidence that glycosylation of H5 HA may affect immunogenicity includes the fact that while antibody titres are higher with Sf9 insect cell-derived HA, neutralisation and HAI titres are much higher with CHO mammalian cell-produced HA [53] – although this does not seem to have been a problem with plant-produced H5 HA VLPs.

An illustration of the speed and scalability of transient expression in *N benthamiana* for emergency response to novel influenza virus outbreaks was shown recently by Medicago Inc., who produced grams of cGMP-grade plant-made H7N9 vaccine, as HA-only VLPs, in response to the outbreak in humans of that very severe influenza virus in China in 2013. The first vaccine lots were available only 19 days after the company accessed the H7 HA gene cDNA sequence, and as little as 3 μg in one dose of the H7 VLP vaccine administered with or without GLA (glucopyranosyl lipid A) adjuvant elicited high antibody titres in mice [54].

In 2012, accounts of Phase 1 trials of both H1N1pdm HA-derived (A/California/04/09) and HPAI H5N1 (A/Indonesia/05/05) HA-derived products were published by researchers from what is now the Fraunhofer Center for Molecular Biotechnology in Delaware, USA (http://www.fraunhofer.org/MolecularBiotechnology), following proof that they could use a TMV-based transient expression system to produce both proteins [48]. Recombinant HA sequences both included KDEL ER retention and 6xHis affinity purification sequences at their C-termini and were purified using detergent-containing buffers, Ni-Sepharose and hydrophobic interaction and anion exchange chromatography columns, at scales up to 50 kg of *N benthamiana* biomass under cGMP. Mouse immunogenicity studies demonstrated that intramuscular (IM) injection of 1 μg of H1 HA or 5 μg of H5 HA protein adsorbed to 0.3% Alhydrogel as adjuvant elicited serum HAI antibody responses with titres ≥1:40 in at least 67% of vaccinated mice, with significant enhancement of immunogenicity and consequent dose-sparing afforded by use of the adjuvant. In rabbits, two IM doses of 45 μg of H1 HA plus Alhydrogel elicited HAI titres of ≥1:40 in 100% of animals, while two doses of 90 μg of H5 HA were required for the same titres in 80% of rabbits. Results in ferrets were similar, with lower responses to the H5 HA. Conclusions from this study were that both antigens were safe and immunogenic, and suited to human trial.

In contrast to the preclinical results, however, testing of the H5 HA vaccine in human volunteers showed no enhancement of immunogenicity by Alhydrogel: 15 and 45 μg doses with Alhydrogel adjuvant, and at 90 μg dose with and without Alhydrogel, were administered in a two-dose regimen three weeks apart; the highest responses were in the 90 μg unadjuvanted group [55].

The H1 HA vaccine antigen was tested with sera derived from human volunteers vaccinated with a conventional AS03 adjuvanted pdmH1N1 vaccine in order to assess its vaccine potential [56]. It was recognized strongly by serum antibodies and antibody-secreting cells from vaccines. Additionally, there was good correlation between results obtained using the plant-made antigen and the conventional vaccine antigen both by ELISPOT and by intracellular cytokine staining assays. The conclusion was that the candidate H1 HA had good vaccine potential, but needed a good adjuvant for use in a clinical trial. This has been done recently [57], in a first-in-human Phase 1 study with H1 HA given twice at dose levels of 15, 45 and 90 μg with and without Alhydrogel, in healthy adults 18–50 years of age. The highest seroconversion rates, measured by HAI (78%) and virus MN assays (100%), were in the 90 μg non-adjuvanted vaccine group after the second vaccine dose.

Thus, results from these two plant-made monomeric candidate HA vaccines are similar, with high doses being required to obtain suitable responses, and – unlike the Medicago trial - no obvious effect of using Alhydrogel adjuvant. It is also questionable whether vaccine antigens containing 6xHis tags would be licenced in a final product.

Another product from this group, however, has more promise: this is an engineered soluble trimeric HA (H1N1pdm) with a heterologous trimerisation motif, which induced serum antibodies active in HAI assays as well as protective immunity in mice given a lethal virus challenge. As may have been expected, the effective doses of the trimeric HA were much lower than previously required for the monomeric HA described above [58]. Further exploration of enhancing the immunogenicity of the H1N1pdm monomeric HA was also promising: this entailed doubly-adjuvanting the antigen with silica nanoparticles (SiO_2_) and the mucosal adjuvant candidate bis-(3′,5′)-cyclic dimeric guanosine monophosphate (c-di-GMP) [59]. Mice were vaccinated intratracheally, which resulted in systemic humoral immune responses, and induction of a strong mucosal immune response with antigen-primed T-cells in the lungs.

**Figure 1:**
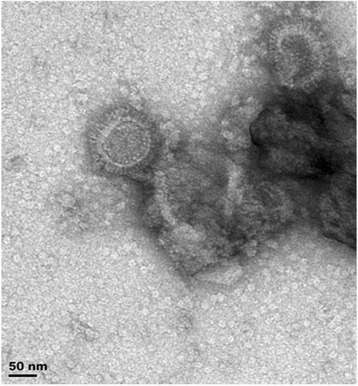
**Transmission electron micrograph of virus-like particles composed of Influenzavirus A H5N1 haemagglutinin (HA) and plant lipids, produced by transient expression of the H5 HA via agroinfiltration of** ***Nicotiana benthamiana*** **leaves with recombinant** ***Agrobacterium tumefaciens*** **.** Full-length HA was produced using the pTra-ERH vector modified for apoplastic secretion, as described by Mortimer et al. [43]. Particles were eluted by gentle centrifugation from leaves that had been vacuum infiltrated with phosphate buffered saline pH 7.4, then cut into strips and rolled up for insertion into centrifuge tubes.

While biofarming is often touted as being ideal for vaccine and other pharmaceutical production for the developing world, there is very little actually published from developing countries on use of the technology for their own purposes. One object example comes from my own lab in South Africa, where we explored the prospects of making emergency response pandemic influenza vaccines [43]. We were successful in making high-yield candidate subunit vaccines via transient agroinfiltration-mediated expression in *N benthamiana* from both a full-length (up to 130 mg/kg FW) and a soluble version (up to 675 mg/kg FW) of the H5N1 A/Viet Nam/1194/2004 HA, via a human codon-use optimization of the HA gene, and showed that while both proteins elicited usable titres of antibodies in mice and chickens by HAI assays, the full length protein was significantly better. Subsequent work showed that the full-length H5 HA could be extracted as semi-pure VLPs from the apoplastic space of unmacerated *N benthamiana* leaves via buffer infiltration and gentle centrifugation of cut leaves rolled into strips (H Inderthal, II Hitzeroth and EP Rybicki, unpublished) (Figure 1).

A number of other different plant-produced influenza vaccine candidates are at earlier stages of development, but show promise: these include HA protein attached to a number of different partner molecules, as well as candidate “universal vaccines” based on the highly conserved M2e or M2 ion channel protein ectopic domain.

Chemical conjugation of plant-made monomeric H1N1 A/California/07/09 HA to the surface of Tobacco mosaic virus (TMV) virions produced a vaccine which elicited a potent antibody response as measured by HAI, and allowed dose-sparing in mice, with a single 15 μg IM dose being sufficient to protect all mice tested, if given with either alum (Alhydrogel) or a squalene oil-in-water based adjuvant (Addavax) adjuvant [60]. The TMV carrier is also unique in that it harnesses dendritic cell (DC) uptake and subsequent activation to stimulate effective antigen presentation, which influences the quality of the subsequent protective immune response. Using TMV as an antigen carrier allows repeated boosting without loss of immune activation to the partner antigen, even when anti-TMV antibodies were already present: in fact, prior exposure to TMV potentiated the response to HA. This group was able to produce 500 g of monomeric HA protein at pilot scale in less than a month – a good advertisement for the potential of this technology for an emergency response vaccine capability.

Another interesting approach was to make an elastin-like polypeptide fusion with a stabilised soluble trimer-forming H5N1 HA (ELPylated H5 HA, or H5-ELP): production was enhanced by ELPylation, and the molecules could be easily purified by the characteristic inverse transition cycling technique, and elicited neutralising antibodies in mice which bound plant-produced H5 HA VLPs and inactivated egg-produced virus [61].

Production of other influenza virus antigens has been limited, with the only other protein investigated to any extent being the M2e peptide, or ectopic domain of the M2 ion channel protein: this has considerable potential as a universal vaccine candidate, as it is highly conserved between all influenza A viruses. The insertion of a M2e_2–24_ peptide in two different locations in the Human papillomavirus type 16 VLP-forming L1 protein has been reported [62], as has the use of surface display on a filamentous VLP-forming potexvirus CP [63], albeit without preclinical evaluation. However, a more recent investigation that used surface display on TMV virions was more noteworthy in that synthetic M2e peptides derived from a H1N1 virus inserted in between TMV U1 CP residues 155 and 156 were well displayed on virions. The candidate vaccines elicited high antibody titres in IM immunised mice, and were 100% protective against 5 LD50s of homologous challenge virus (A/PR/8/34), and 70% of heterologous (A/California/04/2009) challenge [64].

While plant-made influenza viruses in general, and HA-based vaccines in particular seem to be products with significant potential, there are still some avenues to explore – such as the expression and testing of neuraminidase (NA), matrix protein (M1) and nucleoprotein (NP), all of which can play a role in immunity to and recovery from influenza virus infections, and which could constitute valuable additions to the plant-made influenza vaccine armoury.

### Papillomavirus vaccines

Human papillomavirus (HPV) vaccines based on VLPs made by recombinant expression and assembly of the major capsid protein L1 are the latest blockbuster viral vaccines, with annual sales of both Merck’s yeast-made Gardasil and GSK’s insect cell-made Cervarix VLP-based vaccines close to or higher than US$1 billion as of 2012 [65]. Gardasil targets the two most prevalent HPVs found in 70% of cervical cancer cases – HPV-16 and HPV-18 – as well as the two most common genital wart-causing viruses, HPVs −6 and −11, while Cervarix contains only HPVs −16 and −18. While the global impact of the target disease is in the developing world – in 2001 women in developing countries accounted for ~85% of both annual cases of cervical cancer (~500 000 cases worldwide) and annual deaths from cervical cancer (~300 000 worldwide) [66] – the main market for the vaccines is in the developed world, largely due to their expense. While this is changing to some extent, with a number of governments negotiating favourable tenders for supply of HPV vaccines for EPI programmes, it remains a problem – and the imminent release of next-generation products with wider coverage – mixtures of more types of HPV L1, or chimaeric L1 vaccines including all or part of the minor capsid protein L2, or L2-based vaccines - will do nothing to fix this problem [67,68].

Another problem, as yet unaddressed by available vaccines, is therapeutic vaccination for those already infected with high-risk HPVs, or who have severe genital warts: neither Gardasil nor Cervarix has any effect on established infections, and neither will the new wider coverage VLP-based vaccines if they elicit the overwhelmingly humoral antibody responses seen so far [68]. Experimental vaccines based on the high-risk HPV oncogenes E6 and E7 have shown some promise in animal models; however, given that most of the burden of cervical disease as well as cancer is in developing countries [69], the problem of cost and small market size will again be a problem should any licenced vaccines be developed.

The plant-based production of Human papillomavirus vaccines has had quite a long history, that has been well reviewed up to 2010 for all HPV vaccines [10], and 2013 specifically for VLPs [9]. Briefly, the important early landmarks in farming of prophylactic papillomavirus vaccines are the proof that transgenically-expressed HPV-16 or HPV-11 L1 can assemble into immunogenic VLPS, albeit at low yield [70-73]; proofs of efficacy of a plant-made Cottontail rabbit papillomavirus (CRPV) L1-based vaccine [74] and Rabbit oral papillomavirus or CRPV L2 peptides surface-displayed on rTMV vaccines [75]; very high yields of HPV-16 L1 and VLPs via agroinfiltration-mediated transient expression [76] or via transplastomic (chloroplast) expression [77]. An interesting approach was shown in a study that used transplastomic plants to express a HPV-16 L1 mutated so as to form only pentameric capsomers rather than particles, fused to the *Escherichia coli* heat-labile enterotoxin subunit B (LTB) as a built-in adjuvant [78]. The resultant protein accumulated to 2% of total soluble protein (TSP), and had all the correct epitopes and biochemical activity of both parent molecules, indicating that it could be a viable vaccine candidate.

One of the few investigations to look at non-genital HPV L1s was a study of plant expression of the L1 of HPV-8, a high-risk cutaneous papillomavirus associated with epidermodysplasia verruciformis and non-melanoma skin cancer in immunocompromised people [79]. The expressed protein formed VLPs *in planta*, albeit at relatively low yield - and only if the 22aa C-terminal nuclear localisation signal was removed. This is similar to what was found for HPV-11 L1 [73], and in contrast to results for HPV-16, where removal lowered yield [76]. In only the third investigation of expression of the L1 of a non-human papillomavirus, reasonably high levels of protein (183 mg/ml biomass) were achieved for transient agroinfiltration-mediated expression of Bovine papillomavirus type 1 L1 [80]. The protein formed VLPs, albeit only T = 1 30 nm particles rather than the 50 nm T = 7 native structure, which were nevertheless highly immunogenic in rabbits.

The only documented expression of the minor papillomavirus capsid protein L2 was from our laboratory (reported in [81]): this was HPV-16 L2, human codon optimised, and expressed to levels of ~30 mg/kg – which we noted at the time could be useful, as the protein is present in virions at a maximum ratio of 1 L2 molecule per L1 capsomere, and given L1 yields that are ~10–20-fold higher, co-expression of the proteins could result in efficient L1 + L2 VLP formation.

Candidate therapeutic vaccines based on the HPV-16 oncoprotein E7 have been investigated in some detail too: the first expression of the protein in plants – transient expression in *N benthamiana* via a recombinant Potato virus X-derived vector - was accompanied by a proof of efficacy in a mouse model, with protection from tumour development caused by the HPV-16 E7-expressing C3 cell line accompanied by strong cytotoxic T-cell responses [82]. The same group went on to show 5-fold enhanced expression of E7 by targeting of the protein to the secretory pathway [83]. The possibly immune-enhancing properties of *N benthamiana* extracts indicated in previous work were further explored, with proof of immunomodulatory activity on human monocyte derived dendritic cells (MDDCs) [84]. Possibly in response to concerns about using an unmodified oncoprotein as a vaccine, they engineered mutations that abolished interaction with cell cycle governing proteins (E7GGG), and showed that a plant-produced fusion product of this with the beta-1,3-1,4-glucanase (LicKM) of *Clostridium thermocellum* elicited the same protective response [85,86]. Experimental scale-up of production of this fusion protein as a therapeutic vaccine was achieved with 50% final yield (at 100 mg/kg biomass) of protein at 99% purity, by means of metal-ion affinity chromatography and gel filtration [87].

An interesting fusion of prophylactic and potentially therapeutic HPV vaccines was an investigation of the feasibility of producing chimaeric HPV-16 VLPs with L1 fused to a string of cytotoxic T-lymphocyte epitopes from HPV 16 E6 and E7 proteins, in transgenic tomato plants (*Lycopersicon esculentum*) [88]. While expression levels were low, VLPs were formed in planta and elicited both anti-L1 neutralising antibody and anti-E6/E7 CTL responses. A development from this work was the use of the chimaeric VLPs made in tomato plants for detection of HPV-16 specific antibodies in patients with grade 1 cervical intraepithelial lesions (CIN 1) [89], pointing up the potential use of plant-made products as inexpensive reagents.

New developments in the area of mainly prophylactic or VLP-based vaccines are limited to just a few studies, largely involving chimaeric L1 molecules or fusions of L1 to other partners. However, one recent interesting variant to the classic HPV L1 VLP approach was one in which HPV-16 L1 was co-expressed with *E coli* LTB in doubly transgenic tobacco (*N tabacum*) plants to reasonable yield (~0.3% TSP), and extracts were used to orally immunise mice (4x over 52 days) [90]. Combination with LTB dramatically increased intestinal mucosal IgA responses to L1 as well as L1-specific splenic cell proliferation and the expression of IFN-γ and IL-4 in CD4+ T cells and spleen cell supernatants.

A study of chimaeric HPV-16 L1 molecules from our group [91] investigated neutralising antibody epitopes derived from HPV-16 L2 comprising amino acid residues 108–120, 56–81 or 17–36 substituted into the C-terminal helix 4 (h4) region of L1, from amino acid 414, following evidence that these were the most effective in terms of eliciting a wider spectrum of cross-neutralising antibodies to high-risk HPVs [92-95]. Following previous experience with L1, we used a human codon-use optimised L1 gene with similarly-optimised inserts, transiently expressed in *N benthamiana* via agroinfiltration, and targeted for import into chloroplasts. All chimaeras were very highly expressed, with yields of up to 1.2 g/kg plant tissue. The L1 chimaera containing L2 amino acids 108–120 (L1:L2_108–120_) was the most successful candidate in that it assembled into small VLPs (~30 nm), and elicited anti-L1 and anti-L2 responses in IM immunised mice, and immune sera neutralised homologous HPV-16 and heterologous HPV-52 pseudovirions. The other chimaeric L1s did not form distinct particles, and elicited significantly weaker humoral immune responses for the same dose of protein. The L1:L2_108–120_ particle formation was in contrast to repeated previous expression of this and the other chimaeras in insect cells, where only loose aggregates of capsomers were formed for L1:L2_108–120_ [95,96].

Another investigation of the potential of the L2_108–120_ epitope was undertaken using a fusion of the peptide to the N-terminus of the Potato virus X coat Protein (PVX CP) for surface display, expressed via a recombinant PVX vector in *N benthamiana*: expression levels of up to 170 mg/kg biomass were obtained, and the chimaeric protein elicited anti-L2_108–120_–reactive antibodies after SC injection or tattoo administration [97].

E7-based vaccines have continued to be investigated, with a number of approaches yielding promising results. A useful crossover approach mixing potentially prophylactic and therapeutic vaccines was that of the same group who developed the HPV-16 L1 chimaera with a string of T-cell epitopes from HPV 16 E6 and E7 fused to its C-terminus [98]. These workers immunised C57BL/6 mice with the antigen, and could demonstrate persistent anti-L1 IgG antibodies for over 12 months, with good neutralising activity. There was also efficient long-term protection from tumour growth induced by HPV-16 E6/E7-expressing TC-1 tumour cell challenges, and significant tumour reduction (57%) in animals with established tumours given therapeutic vaccination.

In another use of PVX CP fusions, this time with the E7GGG protein mentioned previously [99], it was shown that while both N- and C-terminal fusions with PVX CP formed long filamentous VLPs when expressed in *E coli*, and expression in *N benthamiana* was at high levels, the latter did not apparently form VLPs. Another production modality for E7GGG was investigated in the form of transplastomic *Chlamydomonas reinhardtii*, a well-characterised unicellular alga [100]. E7GGG was expressed alone or as a His6 or FLAG fusion protein, to levels of 0.12% of TSP. Affinity purification was performed for both tagged forms, and C57BL/6 mice were vaccinated SC with *C reinhardtii* extract or purified protein mixed with QuilA adjuvant. Both specific anti-E7 IgGs and E7-specific T-cell proliferation were shown in mice vaccinated with either inoculum, and tumour protection was shown after challenge with TC-1 tumour cell line expressing E7. The authors note that this was the first successful expression of a soluble E7 derivative in plants, as previously the protein had to be expressed as a fusion product in order to get a usable yield.

Another recent paper that explored the expression of E7 in transplastomic tobacco determined that targeting the protein by means of a transit peptide to the thylakoid lumen - a chloroplast inner compartment with different redox potential from the stroma, among other characteristics – increased production by more than 80-fold [101]. However, these authors did not inactivate the oncogenic potential of the protein, which means it presently has value only as an illustration of a useful yield enhancement technique.

Our group has recently explored the potential of a novel HPV-16 E7-derived gene construct – a synthetic shuffled HPV-16 E7 (16E7SH) that has lost its transforming properties, but retains all naturally-occurring CTL epitopes – for expression in plants as a candidate therapeutic vaccine [102]. The E7SH gene has previously successfully been used as a DNA vaccine in a mouse tumour model, eliciting potent cellular and humoral immune responses, including tumour protection and regression [103]. We fused the gene translationally to one encoding the Zera® peptide, a self-assembly domain of the maize gamma-zein seed storage protein that induces the accumulation of recombinant proteins into protein bodies (PBs) within the endoplasmic reticulum in a variety of eukaryotic expression systems [104]. This generally allows stabilisation of recombinant proteins, as well as enhanced accumulation and far easier purification. While E7SH alone was expressed at only a low level by agroinfiltration-mediated transient expression in *N benthamiana*, high-level expression of E7SH-Zera was achieved, with a maximum of 1.1 g/kg biomass, and the resultant protein bodies could be easily purified. Immune responses comparable to the E7SH DNA vaccine were demonstrated in mice, with specific humoral as well as cell-mediated immune responses, and significant tumour regression in vaccinated mice with pre-existing tumours. Interestingly, simply mixing Zera-only PBs and 16E7SH also enhanced immune responses, indicating an independent adjuvant activity for the Zera® component. Moreover, use of the E7SH-Zera gene as a DNA vaccine also resulted in increases in IFN-γ levels of mouse splenocytes compared to mice inoculated with the 16E7SH gene, a trend that was also observed for Granzyme B ELISPOT assays as well as in chromium release assays. We feel we have demonstrated proof of efficacy in a mouse tumour model of a novel HPV therapeutic vaccine candidate, which should be easy and cheap to produce and purify. For further development of this vaccine, a DNA vaccine prime followed by matched protein boost might be ideal in order to achieve further enhancements in immunogenicity.

The overall prospect for plant production of both prophylactic and therapeutic vaccines against HPV infections and disease appears bright: gold-standard VLP-based vaccine candidates can be made at high yield via transient expression, as can viable therapeutic vaccine candidates; efficacy has been demonstrated in animal models for both types of vaccine; early pipeline research appears to show the feasibility of making dual-purpose vaccines that may both prevent infection and hasten recovery from infection.

### HIV vaccines

There is little need to either reiterate the need for vaccines for Human immunodeficiency viruses (HIV) in general, or of the predominant HIV-1 in particular; there is also little need, in the face of a plethora of literature, to detail the approaches to, or problems inherent in, the development of said vaccines [105,106]. As could be expected, HIV vaccines were an early target for plant expression studies, with a variety of targets: indeed, HIV-1 p24 capsid protein was expressed successfully in transgenic tobacco, albeit at low yield (0.35% of TSP), as long ago as 2002 [107]; the suitability of transgenic maize as a production platform for oral delivery of HIV vaccines in seed extracts was tested using SIV major surface glycoprotein gp130 [108]; a novel gp41-derived molecule incorporating a CTB fusion as polymerising agent and adjuvant was produced via agroinfiltration-mediated expression in *N benthamiana* [109] and shown to produce mucosal and serum anti-membrane proximal region (MPR) antibodies in mice after mucosal prime-systemic boost immunisation [110]; a Tat monomer was produced in spinach via rTMV as a potential oral vaccine [111]; epitopes derived from HIV-1 Gag and Env were fused to HBsAg and expressed as VLPs in transgenic tomato fruit [112]; various Gag-derived antigens (p24, p41, p55) were produced in transgenic tobacco and via rTMV in *N benthamiana* for investigation of their utility as CTL-inducing immunogens by our group [113]; HIV-1 Nef was produced in *N benthamiana* using an agroinfiltration-mediated transient expression system [114]. The field was comprehensively reviewed recently [115], so this review will be limited discussion of major advances, and more recent work.

While many and varied antigens have been produced in various types of plant using a variety of expression systems, there have been few systematic investigations of antigens regarded as central to mainstream HIV vaccine production – that is, full-length Gag and Env – and no clinical trials of any candidate vaccine products, although plant-produced anti-HIV MAbs have gone to Phase 1 trial [116]. The most successful expression of full-length Pr^55^ Gag precursor polyprotein to date was done with subtype B HIV-1 Gag in transplastomic tobacco, with yields of enveloped ~100 nm diameter VLPs of up to 400 mg/kg biomass [117]. This was close to 10 000-fold more than a previous best by our group, with VLPs produced in transgenic tobacco [81], and represents a viable yield for a product with serious vaccine potential: recent evidence that lack of progression to AIDS is linked to strong CTL responses to Gag [118-120] reinforces the need for vaccines that can target such responses, and Pr^55^ Gag VLPs are potent elicitors of CTL, especially when used as a protein boost to a heterologous prime in primate models [121,122]. A recent paper details how HIV-1 p24 antigen expressed transgenically in either *Arabidopsis thaliana* or *Daucus carota* showed a priming effect in mice fed whole plant material, eliciting humoral immune responses detected as serum anti-p24-specific IgG after an intramuscular purified p24 protein boost [123]. It is interesting that dose-dependent antigen analyses using transgenic *A. thaliana* showed that low p24 antigen doses were superior to high doses.

A new plant-made HIV multiantigen that makes use of VLPs is “…enveloped particles… consisting of Gag and a deconstructed form of gp41 comprising the membrane proximal external, transmembrane and cytoplasmic domains (dgp41)” [124]. This combines the proven qualities of Gag VLPs as self-adjuvanting potent humoral and cellular response-inducing immunogen with an envelope protein comprising the membrane proximal external region (MPER) of HIV gp41, which is important in infection processes and in eliciting broadly neutralising anti-HIV antibodies. Both the *gag* and *gp41* genes were extensively deconstructed in terms of removal of potential methylation sites, and cryptic splice and polyadenylation sites, and plant codon-use optimised. Gag was expressed constitutively in transgenic *N benthamiana* plants under control of the CaMV 35S promoter, to levels of ~22 mg/kg biomass. Transgenic plants were then infiltrated with Agrobacterium transformed with a deconstructed MagnICON replicating vector expressing the dgp41 envelope protein that was targeted to the apoplast by means of a barley alpha-amylase signal peptide. Coexpression allowed >2-fold greater accumulation of both proteins, with dgp41 reaching ~9 mg/kg. Iodixanol density gradient centrifugation indicated that the coexpressed proteins cosedimented in a denser fraction than for pgp41 when expressed alone, and similarly to Gag-only fractions that were known to contain VLPs – and indeed, characteristic ~100 nm diameter enveloped VLPs were seen in extracts and *in situ* in plant leaf sections, and could be shown to bud into the medium from protoplasted co-transfected cells. I believe these authors are not exaggerating in their claim that “These findings provide further impetus for the journey towards a broadly efficacious and inexpensive subunit vaccine against HIV-1”, given that their vaccine candidate includes both the whole of Gag, and a popular Env-derived target antigen.

The expression of full-length Env or even of gp120 in plants has been an elusive target: there is only a single published account of plant-based expression of HIV-1 Env, and despite this being highly successful, it was only as an adjunct to the demonstration of rapid and high-level transient production of functional HIV broadly neutralising monoclonal antibodies [125]. The Env was the HIV 89.6.P gp140ΔCFI envelope developed by the Vaccine Research Centre, NIH, and was expressed both in stable transgenic SR1 *N tabacum* and transiently via agroinfiltration in *N benthamiana*, as KDEL-tagged and native forms, for ER retention or secretion and consequent differential glycosylation. The gp140 was successfully expressed – to ~80 mg/kg biomass – and purified by leaf homogenization and clarification, followed by *Galanthus nivalis* agglutinin (GNA) lectin column affinity and DEAE ion exchange chromatography. As expected, ER-retained and secreted forms of gp140 were differentially glycosylated, with the former containing only OMT glycans (no complex glycans), and the latter with only low percentages of complex-type N-glycans. This low percentage of complex glycans is similar to the high mannose glycans on virion-associated HIV-1 Env, which differs from the higher percentage of complex glycans on Env produced in mammalian cell lines. The plant-made Env also bound HIV-1-specific MAbs produced either in CHO cells or also produced in plants, indicating no significant difference between it and mammalian cell-produced Env. This is the first convincing evidence for production of a HIV-vaccine-relevant Env molecule in plants, which, together with the evidence for Gag-based VLPs including Env derivatives from the previous work, indicates that true HIV VLPs could be produced in plant systems.

One other Env-related production modality in plants that could be of commercial interest for HIV as well as for other viruses of humans and animals is revealed in a patent filed by Medicago Inc.: this details the use of chimaeric constructs of “ectodomains” from enveloped virus trimeric surface proteins – such as retroviruses, rhabdoviruses, herpes-, corona-, paramyxo-, pox- and filoviruses - fused to an influenza virus HA protein transmembrane domain and cytoplasmic tail, in order to produce VLPs similar to the HA-only VLPs previously mentioned [126]. Their HIV Env construct was a fusion of various portions of the ConS ΔCFI gp145 - an engineered Env lacking the gp120-gp41 cleavage site, the fusion peptide, an immunodominant region in gp41 and the cytoplasmic tail (CT) domain [127] – and the transmembrane (TM) and CT domains of the HA2 portion of either the H3 or the H5 HA molecule. In the words of the patent, “Although native HIV Env protein poorly accumulates in plants, a chimeric HIV Env protein, fused to a transmembrane (TM) and cytoplasmic tail (CT) domains from influenza HA accumulates at high level, and buds into HIV VLPs in absence of core or matrix protein, in plants”. This could provide a genuinely novel source of HIV Env antigens of enhanced immunogenicity, especially for use as a boost vaccine for heterologous prime-boost vaccination regimes.

### Bluetongue virus vaccine

One of the more exciting success stories in veterinary biofarming in recent years is undoubtedly the proof of efficacy of a plant-made VLP-based vaccine for Bluetongue virus (BTV), a muticomponent dsRNA-containing orbivirus in the family *Reoviridae* [128]. This project was a part of the EU FP7 Plant Production of Vaccines (PlaProVa) initiative (http://www.plaprova.eu/), whose activities to do with VLP-based vaccines are partly reported on here [129]. Bluetongue disease is a relatively newly emerged problem in sheep and goats in northern Europe [130], and is almost certainly a result of climate change affecting the distribution of the Culicoides midges that transmit it [131]. Vaccines are seen as an essential part of disease control: however, unlike the case in endemic areas such as South Africa where attenuated live vaccines are used routinely, concerns about vaccine safety and the possible emergence of new strains of virus because of genomic reassortment with live vaccine strains, have resulted in a push for the development of recombinant protein-only vaccines [132].

It has been established that recombinant expression of BTV proteins in insect and other animal cell culture systems results in a variety of structures being formed: expression of VP3 alone produces subcore-like particles (SCLPs); VP3 and VP7 together produce core-like particles (CLPs); expression of these plus VP5 and VP2 produces authentic VLPs [133,134]. Building on evidence that these VLPs are protective in sheep challenged with live virus [135], the PlaProVa group investigated whether it was possible to do the same using plant-produced antigens. Gene constructs were based on the Netherlands NET2006/04 strain of BTV-8, and sequences for VP2, VP3, VP5 and VP7 genes were *Nicotiana* codon-use optimised. The genes were cloned for expression into pEAQ-*HT*, an expression vector containing the Cowpea mosaic virus (CPMV)-derived HT or HyperTrans translational enhancer sequence from RNA2 [136]. Interestingly, infiltration of *N benthamiana* plants with either VP3 and VP5 alone produced necrotic symptoms; if these were combined with the other proteins, no necrosis was observed. Simple screening of clarified extracts by density gradient ultracentrifugation showed that expression of VP3 alone, or of VP3 + VP7, or of all four together, resulted in virion proteins co-sedimenting as high-MW aggregates. Some problems with stoichiometry resulting from relative overexpression of VP3 – an over-accumulation of SCLPs - were addressed by use of vectors expressing more than one protein: VP2 and VP5 were expressed via a dual HT vector, with VP3 and VP7 together on another vector with VP3 expression being detuned by use of a non-HT-containing 5′ UTR. This had the result of down-regulating the formation of CLPs, and a significant shift in the equilibrium towards VLP formation. Total BTV-8 protein yield by use of these vectors was >200 mg per kg biomass; the final yield of gradient-purified VLPs was ~70 mg per kg – gratifyingly high, given a vaccine dose (see below) of 50 μg VLPs/dose.

Testing of the plant-made antigens was done in sheep: immunogenicity was assessed by injecting two sheep with 20 μg of VLPs mixed 1:1 (v/v) with Freund's incomplete adjuvant, and boosting at 21 and 42 days. Serum collected 18 days after first boost was positive for BTV antibodies using a commercial ELISA test kit, and serum from day 56 final bleed reacted with all four structural proteins in western blots, with strongest reactivity toward the major immunogenicity determinant (VP2) and the most abundant structural protein (VP7). Efficacy was tested by injecting four groups of five sheep with either 50 μg VLP or 200 μg CLP mixed 1:1 (v/v) with Montanide ISA70 VG adjuvant, or 5 × 10^4^ TCID_50_/mL commercial live attenuated BTV-8, and boosting on day 28. Animals were challenged with 1 mL infected sheep blood containing live BTV-8 on day 63, and clinical reactions monitored for 2 weeks. Plant-produced VLPs had an identical protective efficacy profile as assessed by clinical reaction index (CRI) as the live attenuated, BTV-8 vaccine, while plant-produced CLPs were poorly protective. Sera from both the VLP and the live attenuated vaccine group showed high serum neutralization titres after day 28; however, VLPs induced high antibody levels only after booster injection, whereas neutralising antibodies were elicited by the attenuated vaccine as soon as 7 days after vaccination. Plant-produced CLPs offered partial protection against live virus challenge, similar to insect cell–produced CLPs.

The results of this investigation are significant for biofarming and vaccinology in particular for a number of reasons. First, as a general finding, it showed it is possible to reasonably easily produce a multi-protein complex with varying stoichiometry: such an approach could also be applied to other complex viruses, such as the related African horsesickness virus, human rotavirus, or picornaviruses such as poliovirus or foot and mouth disease virus. Second, and for BTV in particular, the ability to produce high levels of properly-assembled VLPs that are protective in sheep is a valuable proof of concept and of efficacy for a vaccine against an important emerging disease. Additionally, the timescale of mere days for production following preparation of the agroinfiltration inoculum means that it is possible to respond quickly and potentially locally to outbreaks of emerging disease.

### Dual-use or “One Health” vaccines

The “One Health Initiative” (http://www.onehealthinitiative.com/) is a “…worldwide strategy for expanding interdisciplinary collaborations and communications in all aspects of health care for humans, animals and the environment”, which it hopes to achieve by, *inter alia*, “Joint efforts in the development and evaluation of new diagnostic methods, medicines and vaccines for the prevention and control of diseases across species”. There are a number of obvious viral vaccine targets for this initiative: these are all of the zoonotic viruses that affect domestic and farmed livestock for which there are either unsatisfactory human vaccines or therapeutics, or no human vaccines at all, and high-impact recently emerging viruses with no vaccines for animals or humans. A good example in the former category would be rabies virus; examples for the second would be agents such as Severe acute respiratory syndrome (SARS) and Middle Eastern respiratory syndrome (MERS) coronaviruses (CoVs), West Nile virus (WNV), and Ebola and Marburg filoviruses. Thomas Monath has also recently defined a “one health paradigm” [137] which specifies three frameworks for development and use of vaccines to control zoonoses:

- Framework I vaccines target dead-end human and livestock hosts.
- Framework II vaccines target [mainly arthropod-vectored] infections of domesticated animals as a means of preventing spread to humans.
- Framework III vaccines target wild animal reservoirs.

His example for Framework 1 vaccines would be West Nile virus; for Framework II, Rabies virus, Rift Valley fever, Venezuelan equine encephalitis, and Hendra viruses; for Framework III, reservoir-targeted agents such as oral bait rabies.

Given the obviousness of the targets, it is surprising that very few of them have been explored for their potential as plant-made vaccines. Rabies is one of these, and relatively early on: a chimaera of Alfalfa mosaic virus CP and epitopes derived from glycoprotein G and the nucleoprotein was successfully expressed in plants via two different plant virus-based systems. Parenterally-injected extracts protected mice from challenge, and human volunteers who had ingested rCP-containing plant material produced rabies virus-neutralising antibodies [138]. A full-length synthetic G protein gene – with a plant secretion signal peptide, and ER retention signal – was found to express reasonably well (0.4% TSP) in transgenic tobacco, and purified protein elicited complete protection against virulent intracerebral challenge in intraperitoneally-immunised mice [139].

The development of an effective oral vaccine against rabies has also been reported, with single doses of 50 μg Vnukovo strain rabies glycoprotein G in transgenic maize seed containing the antigen at 1% of TSP, protecting all vaccinated mice from lethal vampire bat rabies virus challenge [140]. Further testing of this vaccine was done in sheep: maize kernels containing different doses of G protein (0.5, 1, 1.5 and 2 mg) were given in a single dose by the oral route, and 2 mg doses elicited a degree of protection comparable to that conferred by the injected commercial vaccine [141]. The authors state that “…this is the first study in which an orally administered edible vaccine showed efficacy in a polygastric model”, which is an important landmark in veterinary and potentially One Health vaccinology.

Two important disease agents that are prime candidates for consideration as One Health vaccines and occur almost exclusively in developing countries, are the tick-borne Crimean-Congo haemorrhagic fever virus (CCHFV) and mosquito-borne Rift Valley fever virus (RVFV), both familial bunyaviruses. CCHFV has the wider distribution, including Africa, Asia, southern and eastern Europe and the Middle East [142], while RVFV is largely restricted to sub-Saharan Africa. However, both viruses are regarded as having emerging potential, with RVFV in particular regarded as having the potential to spread to Europe, Asia, and the Americas [143]. While attenuated live vaccines are available for RVFV, these are regarded as having significant side effects; there are no accepted vaccines for either virus for general use in humans.

A recent study investigated the expression of the two neutralizing epitope-rich CCHFV envelope glycoproteins Gc and Gn in hairy root cultures and in leaves derived from transgenic tobacco plants [144]. Proteins accumulated to levels of 1.8 mg/kg biomass in hairy roots and 1.4 mg/kg in leaves. Separate groups of mice were fed transgenic leaves or roots, or fed the plant material and injected SC with the plant-made proteins, or vaccinated with an attenuated CCHFV vaccine as a positive control. Mice in all the immunised groups had a consistent rise in anti- Gc and Gn IgG and IgA antibodies in serum and faeces, respectively. Mice in the group that was fed and parenterally boosted, however, exhibited a significant rise in anti-CCHFV IgG (titre of 1/32 000 compared to 1/256 for oral-only) after a single boost. Additionally, the plant-purified Gc and Gn proteins reacted with human immunoglobulins in serum from a patient who had recovered from CCHFV infection. The potential for recombinant protein production in plants as a CCHFV vaccine appears obvious, although yields seem low for routine production.

The production of Rift Valley fever virus antigens in plants is much less well studied: there is one PhD thesis that reports expression of a truncated Gn or soluble ectodomain construct and of the nucleoprotein (N) gene in transgenic *Arabidopsis thaliana* [145]. This study chose these two antigens because Gn is the more effective at eliciting neutralising antibodies of the two envelope glycoproteins and the recombinant Gn ectodomain (tGn) is known to be protective, and the N antigen elicits non-neutralising antibodies but also a strong cellular immune response that is partially protective. The N protein accumulated to high levels while truncated Gn protein did not accumulate to levels detectable by western blot. Feeding groups of mice transgenic plant material three times (0, 2 and 4 weeks) containing tGn or ~70 μg of N protein per 4 mice resulted in seroconversion after the second feeding for both antigens, with higher titres (10^3^ – 10^4^) for N compared to tGn (10^2^ – 10^3^). While these results are very preliminary as far as a vaccine goes, they indicate the feasibility of first, using transgenic or transiently-produced antigen in plant tissue as an oral vaccine in animals, and second, of producing antigen for complete or partial purification processes that could result in a human vaccine.

### Anti-viral therapeutic antibodies

A useful recent review details how plant-based antibody products may “…provide lower upfront cost, shorter time to clinical and market supply, and lower cost of goods (COGs)…[and] improvements in pharmacokinetics, safety and efficacy” [146]. As an object example, an important aspect of rabies disease prevention is therapy, given the many people in developing countries who get bitten by suspected rabid animals annually – and a major development in this area is the production of potent rabies-neutralising antibodies in plants, given the prevailing situation of mainly equine-produced sera being in short supply and of variable quality. A consortium of researchers that includes South Africans have recently described the engineering and production in transgenic *N tabacum* plants of both a humanised IgG version of the broadly neutralising murine MAb E559, and the murine version [147]. Purification via agarose protein A/G affinity chromatography yielded 1.8 mg/kg biomass (0.04% TSP) for the chimaeric Mab, and 1.2 mg/kg biomass (0.03% TSP) for the murine Ab. Both antibodies assembled properly, and were equivalent to hybridoma-produced MAbs in neutralising a panel of lyssaviruses that included all the phylogroup I viruses classical RABV, Duvenhage virus, European bat lyssavirus types 1 and 2, and Australian bat lyssavirus, although no neutralisation was seen for the phylogroup II viruses Lagos bat virus and Mokola virus. The efficacy in post-exposure prophylaxis of the humanised Ab was tested in hamsters injected with a lethal dose of CVS-11 strain of virus: the plant-produced antibody was apparently more effective than the commercial human rabies immunoglobulin (HRIG; Rabigam), in that survival for both treatment groups was >50% after 14 days, and zero and 11% for HRIG and plant Ab groups, respectively, after 28 days.

The production of anti-Ebola virus antibodies has also recently been explored in plants: this could yet become an important part of the arsenal to prevent disease in healthcare workers, given that at the time of writing an uncontrolled Ebola haemorrhagic fever outbreak was still raging in West Africa (http://www.who.int/csr/don/archive/disease/ebola/en/), had spread from Guinea to Sierra Leone, Liberia, Nigeria, Spain and to the USA (http://www.promedmail.org/direct.php?id=2823539), and the use of experimental solutions was not only being suggested [148], but was being put into practice.

As background, use of a high-yielding geminivirus-based transient expression system in *N benthamiana* that is particularly suited to simultaneous expression of several proteins had previously allowed expression of a MAb (6DB) known to protect animals from Ebola virus infection, at levels of 0.5 g/kg biomass [149]. The same group also used the same vector system (described in detail here [24]) in lettuce to produce potentially therapeutic MAbs against both Ebola and West Nile viruses [150].

A more comprehensive investigation was reported recently, of both plant production of Mabs and post-exposure prophylaxis of Ebola virus infection in rhesus macaques [151]. Three Ebola-specific mouse-human chimaeric MAbs (h-13 F6, c13C6, and c6D8; the latter two both neutralising) were produced in whole *N benthamiana* plants via agroinfilration of magnICON TMV-derived viral vectors. A mixture of the three MAbs – called MB-003 – given as a single dose of 16.7 mg/kg per Mab 1 hour post-infection followed by doses on days 4 and 8, protected 3 of 3 macaques from lethal challenge with 1 000 pfu of Ebola virus. The researchers subsequently showed significant protection with MB-003 treatment given 24 or 48 hours post-infection, with four of six monkeys testing surviving, compared to none in two controls. All surviving animals treated with MB-003 experienced insignificant if any viraemia, and negligible clinical symptoms compared to the control animals. A significant finding was that the plant-produced MAbs were three times as potent as the CHO cell-produced equivalents – a clear case of plant production leading to “biobetters”. A follow-up of this work investigated efficacy of treatment with MB-003 after confirmation of infection in rhesus macaques, “according to a diagnostic protocol for U.S. Food and Drug Administration Emergency Use Authorization” [152]. In this experiment 43% of treated animals survived, whereas all controls tested here and previously with the same challenge protocol died from the infection.

An article published during the Ebola outbreak [153] detailed the therapeutic use in rhesus macaques of a cocktail of anti-Ebola Mabs called ZMapp. This is described as a successor to MB-003, incorporating components of the ZMAb cocktail developed by the National Microbiology Laboratory of the Public Health Agency of Canada and another antibody mixture, and was developed by Mapp Biopharmaceutical of San Diego with Defyrus of Toronto and manufactured by Kentucky BioProcessing. The cocktail proved able to rescue 100% of macaques when administered up to 5 days post live virus challenge. It was noteworthy that advanced disease in many animals - indicated by elevated liver enzymes, mucosal haemorrhages and generalised petechia - was reversed, leading to full recovery. ZMapp was also found to bind virions of the Guinean variant of Ebola implicated in the present outbreak. The authors claim that “ZMapp exceeds the efficacy of any other therapeutics described so far, and results warrant further development of this cocktail for clinical use”.

In news received during consideration of this article that may vindicate this view, a report quoted as coming from the National Institute of Allergy and Infectious Diseases states that two US healthcare workers who contracted Ebola in Liberia were treated with ZMapp [154]. Despite being given up to nine days post-infection in one case, it appears to have been effective [155]. In later developments, the therapy was also given to another five people, two of whom died. The US government was negotiating at time of writing to produce large amounts of ZMapp as a first-line therapy [156,157]. As an illustration of the scale-up problem facing the manufacturers, in the macaque trial referred to above [151], animals were given three doses of ZMapp at 50 mg/kg intravenously at 3-day intervals. For an adult 70 kg human given the same dose, this would mean a total of 10.5 (3 × 3.5) grams of ZMapp: assuming optimal purified yield of each of the three MAbs individually at 100 mg/kg plant biomass, this would mean 105 kg of *N benthamiana* would be needed to dose just one person optimally. While biofarming may be the most scalable technology for producing MAbs and other therapeutics, this sort of scale requires resources far larger than currently exist.

A novel application of the same technology was also used to produce an Ebola immune complex (EIC) in *N benthamiana*, consisting of the Ebola envelope glycoprotein GP1 fused to the C-terminus of the heavy chain of the humanised 6D8 MAb, which binds a linear epitope on GP1. Geminivirus vector-mediated co-expression of the GP1-HC fusion and the 6D8 light chain produced assembled immunoglobulin, which was purified by protein G affinity chromatography. The resultant molecules bound the complement factor C1q, indicating immune complex formation. Subcutaneous immunisation of mice with purified EIC elicited high level anti-GP1 antibody production, comparable to use of GP1 VLPs [158]. This is the first published account of an Ebola virus candidate vaccine to be produced in plants.

### Future prospects for plant-produced vaccines

The prospects for the increasing use of plants for the manufacture of pharmaceuticals in general look increasingly bright, especially with the recent approval and licensure of Protalix’s carrot cell-produced Gaucher disease therapeutic enzyme glucocerebrosidase, traded as ELELYSO™ (taliglucerase alfa) (http://www.protalix.com/products/elelyso-taliglucerase-alfa.asp). This product was approved by the US Food and Drug Administration for injection in May 2012 for long-term replacement therapy for adults with a confirmed diagnosis of Type 1 Gaucher disease. While it is marketed as a biosimilar to the mammalian cell-produced enzyme, it is in fact a biobetter, given that it is naturally mannosylated – which aids uptake by macrophages, for example – unlike the conventional product, which has to be chemically altered. Other biologics such as therapeutic MAbs for use as anti-HIV agents in microbicides are also getting very close to registration – and the recent experience with anti-Ebola MAbs may well speed the process.

However, while the prospects for animal vaccines in general and possibly human therapeutic vaccines also seem favourable, given an increasing number of proofs of efficacy in this sphere, for prophylactic human vaccines specifically they are not as bright – in the short term, at least. The length and rigour of the human prophylactic vaccine developmental and clinical testing path compared to animal and human therapeutic vaccines are a huge obstacle to commercial production of novel biofarmed vaccines, as is the entrenched investment in conventional technology by Big Pharma. It may be that the technology will find a niche on the fringes of conventional vaccinology, where there is a need for small-scale production of vaccines for orphan diseases or for rapid responses to bioterror-related or emerging viral disease outbreaks. One area where biofarmed vaccines could break through soon could be rapid-response vaccines to novel influenza virus outbreaks: the capacity for such a response has already been demonstrated (see above); it remains to put it to the test in a real-life scenario. Once this happens, there is an increased likelihood of the technology being employed for other agents, such as MERS-CoV, RVFV and West Nile and Chikungunya viruses, where “One Health” principles are important.

## Conclusions

The widespread application of biofarming – or more properly, plant molecular farming – that was anticipated in the 1990s has been long in coming, and there are still cynics that doubt that it will ever happen. However, it is almost certainly the case that there is now enough accumulated evidence of proofs of principle in humans and animals, and of efficacy mainly in animals but in humans too, that its great potential for the cheaper and more rapid and more widely scalable manufacture of high-value biologics and pharmaceuticals can no longer reasonably be denied. In the case of viral vaccines and therapeutics specifically, it has proved possible to routinely make fully-assembled complex VLPs and immunoglobulins that can either elicit potent protective immune responses, or disease-reducing therapeutic effects, as shown in animal models.

The recent history of plant-made HBV and HPV and HIV vaccine candidates in particular has shown huge yield increases for viral antigen production, largely as a result of application of sophisticated transient expression techniques that are rapidly becoming the industry norm. The surprising revelation that it was easy to make high-yield HA-only VLPs for influenza viruses in plants could transform that particular industry; so too the fact that plant-made BTV VLPs are probably as effective as insect- or mammalian cell-made VLPs and probably far cheaper to make, and far safer to make and use than attenuated BTV vaccines.

Thus, it seems a reasonably safe prediction that biofarmed viral vaccines will be approved more widely for at least animal use in the near future, and possibly also for use as therapy for existing infections and emergency response therapeutics and vaccines in humans in the slightly longer term – such as for rabies, and possibly for Ebola. Hopefully, approval for prophylactic vaccines for humans – such as for seasonal influenza – will follow in the slightly longer term.
